# Material Visual Perception and Discharging Robot Control for Baijiu Fermented Grains in Underground Tank

**DOI:** 10.3390/s24248215

**Published:** 2024-12-23

**Authors:** Yan Zhao, Zhongxun Wang, Hui Li, Chang Wang, Jianhua Zhang, Jingyuan Zhu, Xuan Liu

**Affiliations:** 1 School of Mechanical Engineering, University of Science and Technology Beijing, Beijing 100083, China; yanzhao@ustb.edu.cn (Y.Z.); jhzhang@ustb.edu.cn (J.Z.); jingyuanzhu0129@163.com (J.Z.); 2 School of Mechanical Engineering, Hebei University of Technology, Tianjin 300401, China; wangzxoffice@163.com (Z.W.);

**Keywords:** discharge of fermented grains, visual perception, edge detection, point cloud processing, three-dimensional reconstruction

## Abstract

Addressing the issue of excessive manual intervention in discharging fermented grains from underground tanks in traditional brewing technology, this paper proposes an intelligent grains-out strategy based on a multi-degree-of-freedom hybrid robot. The robot’s structure and control system are introduced, along with analyses of kinematics solutions for its parallel components and end-effector speeds. According to its structural characteristics and working conditions, a visual-perception-based motion control method of discharging fermented grains is determined. The enhanced perception of underground tanks’ positions is achieved through improved Canny edge detection algorithms, and a YOLO-v7 neural network is employed to train an image segmentation model for fermented grains’ surface, integrating depth information to synthesize point clouds. We then carry out the downsampling and three-dimensional reconstruction of these point clouds, then match the underground tank model with the fermented grain surface model to replicate the tank’s interior space. Finally, a digging motion control method is proposed and experimentally validated for feasibility and operational efficiency.

## 1. Introduction

Underground tank fermentation is a traditional fermentation process that utilizes round ceramic cylinders buried in the ground as fermentation vessels, possessing irreplaceable artisanal value in Baijiu production. The underground tank not only effectively shields against impurities from the soil, thus preventing the generation of unpleasant odors during fermentation, but also maintains a constant temperature inside the tank, providing an optimal fermentation environment for fermented grains. These favorable characteristics contribute to the production of pure and rich-flavor Baijiu. However, the pottery tank’s hard and brittle texture, coupled with its limited internal workspace, makes the fermented grains susceptible to compression damage when digging out, leading to a decreased alcohol yield. Consequently, Baijiu enterprises predominantly rely on manual methods for discharging fermented grains, inevitably amplifying the drawbacks of labor-intensive processes and low production efficiency inherent in traditional brewing procedures, significantly hindering the automation progress of Baijiu production lines [1,2]. Currently, the studies on discharging fermented grains from underground tanks primarily focus on the structures of the discharge equipment, with relatively limited exploration into automated discharge methods. However, existing devices for discharging fermented grains either damage the raw material particles of fermented grains or leave excessive residues inside the tanks, which fails to meet the working requirements [3]. Therefore, there is an urgent need for the development of an efficient intelligent discharge strategy.

Visual sensing represents the most intuitive method for robots to perceive their environment, with numerous scholars leveraging this technology to investigate automated grasping or excavation methods for various application scenarios. Rath et al. differentiated overlapping flower stalks using a binocular vision system to create three-dimensional models of plants, subsequently guiding a six-axis industrial robot to sever stalks for harvesting [4]. Huang et al. employed a binocular stereoscopic vision algorithm to compute the three-dimensional coordinates of grasp points, developing a visual-guided system for picking phalaenopsis tissue culture seedlings [5]. Leveraging advancements in deep learning, researchers exploit its robust analytical and recognition capabilities to extract enhanced visual features from images, guiding robots towards more precise excavation tasks [6]. Takahashi et al. utilized the CNN to predict the grasping quantity and uncertainty in different regions of RGBD images, selecting optimal picking points [7]. Schenck et al., assuming the known initial states of particles, used ConvNets to predict particle states based on parameters of the robotic arm’s bucket-dumping action [8]. Hu et al. combined convolutional neural networks (CNNs) and residual networks (ResNet) to leverage the strengths of both approaches, creating a hybrid model for multi-object grasping strategy prediction [9]. The YOLO series, a representative first-order detection algorithm, is widely used in object detection due to its real-time performance and simple model structure. Ding et al. proposed a machine-vision-based automatic digging depth control system, employing an enhanced YOLO-v5 algorithm to calculate in real time the length of the grasped garlic roots, and utilizing an STM32 microcontroller to control the digging depth via an electrically operated push rod for expanding and contracting the garlic digging device [10]. Sun et al. proposed a parallel YOLO-GG deep vision network based on YOLO and GG-CNN, which, through experimental validation, demonstrated enhanced efficiency and accuracy in collaborative robot object classification [11]. Lundell et al. introduced a deep network—DDGC—that enables collision-free grasping in complex environments by utilizing information from a single RGBD image [12]. Tong et al. addressed the detection and grasping of objects with weak textures by designing an advanced semantic segmentation algorithm (RTSegNet), which effectively segments transparent and reflective objects using RGB information, thus improving the accuracy of real-time object grasping by robots [13]. These studies indicate that deep-learning-based grasping strategies hold significant promise for enhancing robotic task efficiency and ensuring accuracy. Additionally, some work [14,15] explored fuzzy logic algorithms to enhance the accuracy and efficiency of robotic excavation. Beyond accurate target recognition, determining robotic excavation trajectories and strategies ensures high efficiency, stability, and dynamic adaptability. Huh et al. applied an artificial intelligence module featuring LSTM as the main layer in a trajectory-planning system for buckets, proposing a secure excavation method robust against successive excavations and capable of avoiding collisions with underground obstacles [16]. Jud et al. defined the interaction force trajectories of end-effectors over a single excavation cycle, enabling adaptive excavation motions for diverse terrains, and introduced a large-scale iterative planner for continuous single-pass excavation until achieving the desired ground geometries [17]. Zhao et al. transformed arbitrarily slow and non-smooth human excavation trajectories into topologically equivalent paths, optimizing trajectories with respect to time and acceleration [18]. Yang et al. constrained instantaneous bucket motions and added target-specific constraints to control the excavation volume, proposing a novel optimization-based framework adaptable to diverse terrains [19]. Liu et al. utilized the Direct Configuration Method (DCM) to optimize the trajectory of a Selective Compliance Assembly Robot Arm (SCARA). The experimental results demonstrated that DCM effectively reduces both power consumption and positional errors [20]. Li et al. applied the deep deterministic policy gradient (DDPG) algorithm to solve the complex constraint motion planning of a free-floating dual-arm manipulator, modifying the reward function to account for end-effector self-collision avoidance and velocity constraints. The feasibility of this approach was verified through experiments [21]. During robot operation, in addition to obstacle avoidance, environmental constraints must also be considered. Song et al. used Speeded-Up Robust Features (SURF) to localize targets in the robotic arm’s workspace and designed two safety indices to enhance its safe operation in unknown environments [22]. Chen et al. proposed a collision-free motion-planning method for robots based on potential fields and virtual force constraints. This method uses multiple depth sensors to perceive obstacle positions, treating obstacle points as constraints with virtual forces, and employs a potential field algorithm to generate collision-free motion trajectories [23]. Most of these methods and technologies are largely applicable in open workspaces; however, their feasibility and efficiency in narrow environments such as trenches require further experimental validation.

Due to the fermentation vessel being located underground with poor lighting conditions, it differs significantly from above-ground operations. When discharging the fermented grains, it is crucial to first ensure no contact with the interior walls of the underground tank to avoid damaging the grains, followed by considerations of digging volume and efficiency. Based on a self-developed multi-degree-of-freedom hybrid robot, this paper proposes an intelligent discharge strategy utilizing visual perception. This includes the perception of underground tanks’ positions and fermented grain surfaces, along with motion control methods for discharging fermented grains. This paper is organized as follows: Section 2 provides a brief overview of the hybrid robot’s overall structure, control system, and kinematic analysis of its parallel components, including inverse kinematics and end-effector velocity analysis. Section 3 elaborates on the proposed intelligent discharge strategy, while Section 4 validates its feasibility and effectiveness through experimental verification. Finally, Section 5 concludes the paper.

## 2. Discharge System

### 2.1. The Introduction of Hybrid Discharge Robot

**Figure 1:**
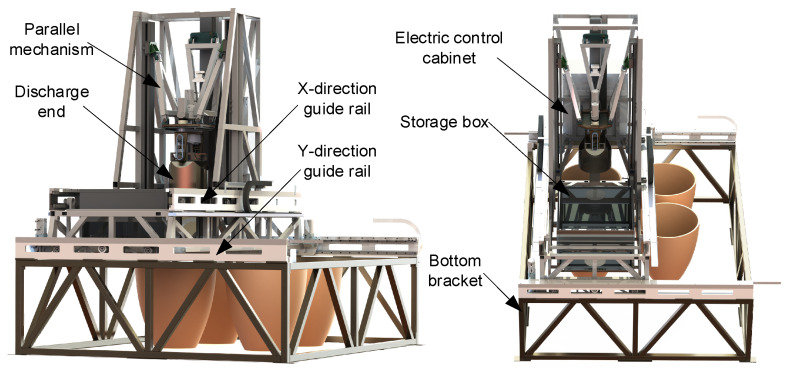
Configuration of the hybrid robot.

In response to the challenges of discharging fermented grains in traditional Chinese brewing techniques, we have developed an intelligent discharge robot to achieve an efficient and accurate discharge operation. As depicted in Figure 1, the overall structure consists of a horizontal-running guide rail mechanism and a 2PSR/1PRS parallel mechanism, with the discharge end mounted on the moving platform of the 2PSR/1PRS parallel mechanism. This PP2PSR/1PRSRR hybrid robot features 7 degrees of freedom. The horizontal-running guide rail mechanism comprises V-type rails, rollers, and helical gear racks fixed on the bottom bracket and bearing frame. Servo motors drive the equipment via the helical gears to achieve XY-axis movement. The 2PSR/1PRS parallel mechanism utilizes rollers mounted on the guide rails and a linear module to control the discharge end’s movement along the Z-axis and swinging in the XY direction. The end-effector is equipped with motors and a TOF depth camera. The motors drive the rotation and flipping of the discharge end for two degrees of freedom, while the Time-of-Flight (TOF) depth camera primarily handles tasks such as underground tank positioning and fermented grain surface detection.

**Figure 2:**
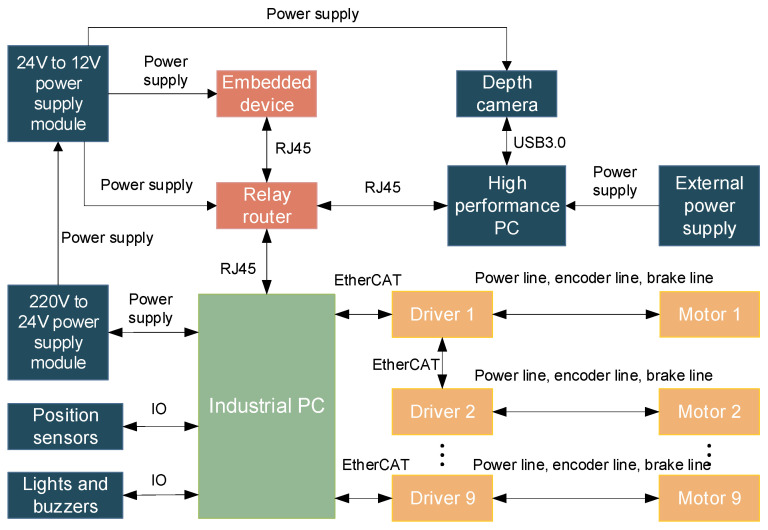
Hardware system framework diagram.

The hardware of the discharge robot mainly consists of an industrial PC, an embedded device, a high-performance PC, a TOF depth camera, servo motors and drivers, power modules, a relay router, and sensors, as shown in the system architecture diagram in Figure 2. The TOF depth camera is used for capturing color images and depth images. The high-performance PC is connected to the depth camera for receiving image data and running visual algorithms. Embedded devices handle position calculation and motion planning. Industrial PCs are connected to position sensors, indicator lights, buzzers, and drivers. They receive motion commands from the embedded devices, convert them into motor motion parameters, and thereby control the robot’s movement.

### 2.2. Analysis of 2PSR/1PRS Parallel Mechanism

#### 2.2.1. Calculation of Degrees of Freedom

**Figure 3:**
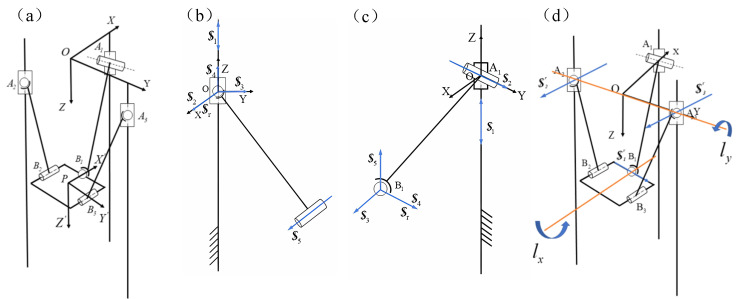
(**a**) Structure schematic coordinate system; (**b**) schematic diagram of the spiral system of PSR branched chain movement; (**c**) schematic diagram of the spiral system of PRS branched chain movement; and (**d**) schematic diagram of the inverse spiral system.

The coordinate systems of the parallel mechanism and the helical systems of each branch are established as shown in Figure 3a, b, and c, respectively, to derive its inverse helical system as illustrated in Figure 3d. Based on the Grassmann line geometry principle [24], the analysis indicates that the moving platform can rotate about the $l_{y}$, rotate about the $l_{x}$, and move in the Z direction, implying that the parallel mechanism possesses three degrees of freedom for the rotation about the X-axis, rotation about the Y-axis, and movement along the Z-axis.

#### 2.2.2. Inverse Kinematics

The dynamic platform of the 2PSR/1PRS parallel mechanism possesses three degrees of freedom, namely, the movement in the Z direction and rotation in the XY plane. Through an analysis of the degrees of freedom, it is evident that, when the end-effector rotates in the XY plane, there are accompanying movements in other degrees-of-freedom directions. Only the z, α, and β parameters are independently controllable. The output of this parallel mechanism is denoted as $T={\left[\begin{array}{ccc} z & \alpha & \beta \end{array}\right]}^{T}$, and the input as $q={\left[\begin{array}{ccc} L_{1} & L_{2} & L_{3} \end{array}\right]}^{T}$. Based on the characteristics of the rotational joints, the links connected to the rotational joints can only move within a plane centered at the rotational joint. Consequently, the trajectory of point $B_{1}$ lies within the OXZ plane of coordinate system {O}, while the trajectories of points $A_{2}$ and $A_{3}$ lie within the $PX^{\prime }Z$ plane of coordinate system {P}, implying that the Y coordinate of point $B_{1}$ in {O} is 0, and the X coordinates of points $A_{2}$ and $A_{3}$ in {P} are 0. This yields three constraint equations:

(1) $$\left\{\begin{array}{l} P_{y}+{\mathrm{PB}}_{X}^{1}\left(c\alpha s\gamma +c\gamma s\alpha s\beta \right)=0\\ L_{2}\left(c\gamma s\alpha +c\alpha s\beta s\gamma \right){+\mathrm{OA}}_{Y}^{2}c\alpha c\gamma -P_{y}\left(c\alpha c\gamma -s\alpha s\beta s\gamma \right)-P_{z}\left(c\gamma s\alpha +c\alpha s\beta s\gamma \right)\\ -{\mathrm{OA}}_{Y}^{2}s\alpha s\beta s\gamma -P_{x}c\beta s\gamma =0\\ L_{3}\left(c\gamma s\alpha +c\alpha s\beta s\gamma \right){+\mathrm{OA}}_{Y}^{3}c\alpha c\gamma -P_{y}\left(c\alpha c\gamma -s\alpha s\beta s\gamma \right)-P_{z}\left(c\gamma s\alpha +c\alpha s\beta s\gamma \right)\\ -{\mathrm{OA}}_{Y}^{3}s\alpha s\beta s\gamma -P_{x}c\beta s\gamma =0 \end{array}\right.$$

Due to the fixed lengths of the connecting rods, the inverse kinematics of the mechanism can be obtained as follows:

(2) li=BiO−AiO i=1~3

where $l_{i}\left(i=1\sim 3\right)$ represents the lengths of the connecting rods, AiO and BiO represent the coordinates of points $A_{i}(i=1\sim 3)$ and $B_{i}(i=1\sim 3)$ in the static coordinate system {O}, respectively.

From Equation (2), it is evident that a direct relationship between the input $q={\left[\begin{array}{ccc} L_{1} & L_{2} & L_{3} \end{array}\right]}^{T}$ and output $T={\left[\begin{array}{ccc} z & \alpha & \beta \end{array}\right]}^{T}$ of the parallel mechanism cannot be determined. Instead, three non-homogeneous linear equations and three constraint equations can be derived. Solving the linear equations enables the inverse kinematics to be obtained.

#### 2.2.3. Forward Kinematics

The PSR/1PRS parallel mechanism belongs to a type of coupling, with limited degrees of freedom. While the mechanism has three inputs, all six end-effector pose parameters can vary, necessitating the correction of the dynamic platform’s position relative to the fixed coordinate system as $T={\left[\begin{array}{llllll} x & y & z & \alpha & \beta & \gamma \end{array}\right]}^{T}$. The virtual workspace of this parallel mechanism is not only influenced by the dimensions of the mechanism structure but also by the characteristics of coupled motion. When iteratively searching along the direction guided by the Jacobian, the mechanism may exceed its virtual workspace, resulting in solution failure.

**Figure 4:**
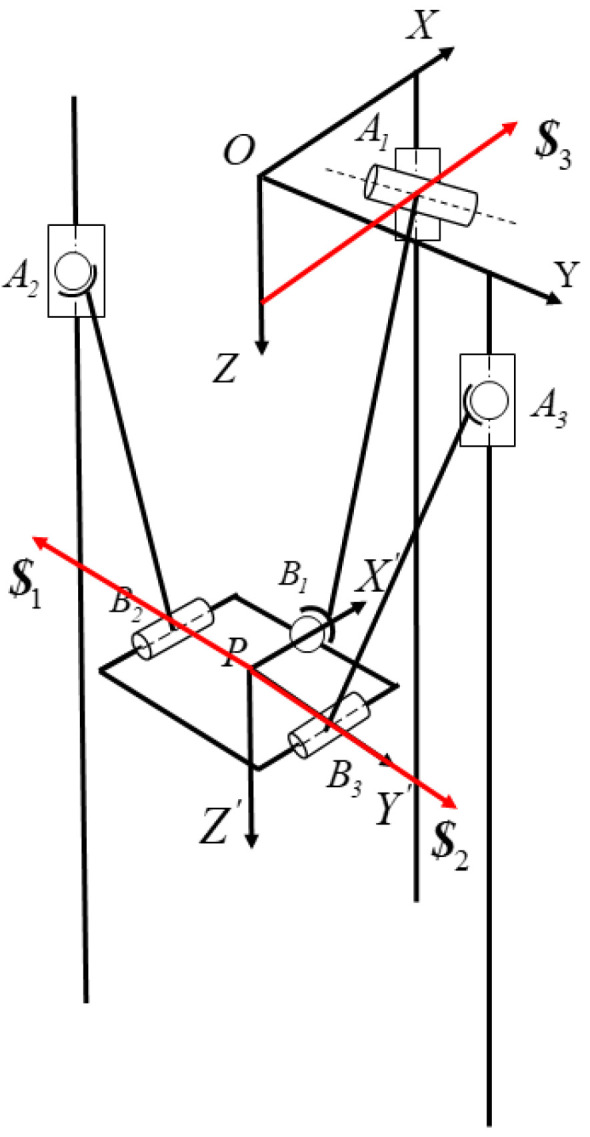
Diagram of the 2PSR/1PRS structure with added virtual branches.

This paper employs the method of the fictitious branch to address the coupling constraint issue. The fictitious mechanism method increases the number of input mechanisms by adding virtual mechanisms, thereby eliminating the coupled motion characteristics of the mechanism’s virtual space. As shown in Figure 4, rotational joints are added at points $A_{1}$, $B_{2}$, and $B_{3}$ as virtual motion pairs for the parallel mechanism, each perpendicular to the original rotational joints at the connection points. The forward kinematics solution process for the parallel mechanism requires the establishment of an inverse kinematics model. Due to the addition of three rotational joints with the fictitious mechanism method, introducing three additional degrees of freedom, it is necessary to include equations for the angles of rotation for these three additional joints.

The rotation angles of each virtual rotational joint are denoted as follows:

(3) $$\theta_{i}=\arccos\left(n_{i}\cdot \left(l_{i}\times A_{i}\right)/\left(\left|l_{i}\times A_{i}\right|\right)\right)$$

where $\theta_{i}\left(i=1\sim 3\right)$ represents the rotational angle of each fictitious rotational joint, $n_{i}\left(i=1\sim 3\right)$ denotes the unit direction vector of the initial position for each virtual branch, $l_{i}\left(i=1\sim 3\right)$ denotes the positional equations of each connecting rod, and $A_{i}$ signifies the coordinates of the connection points.

Post-fictitiously, the mechanism transforms into a 2PSU/1PUS parallel mechanism, featuring six degrees of freedom. The fictitious rotational joints also become inputs, with their generalized input denoted as $q={\left[\begin{array}{llllll} L_{1} & L_{2} & L_{3} & \theta_{1} & \theta_{2} & \theta_{3} \end{array}\right]}^{T}$. Utilizing Newton’s method for iteration [25] and setting $q_{m}={\left[\begin{array}{llllll} L_{1} & L_{2} & L_{3} & 0 & 0 & 0 \end{array}\right]}^{T}$, the forward kinematics solution for the parallel mechanism can be accomplished.

#### 2.2.4. Velocity Analysis of the Discharge End

In the discharging process, the analysis of the end-effector velocity is also a focal point of study. The velocity at the end-effector determines both the digging efficiency and the pouring efficiency. Therefore, the relationship between the velocities of the two motors at the end-effector and the discharge end is analyzed.

**Figure 5:**
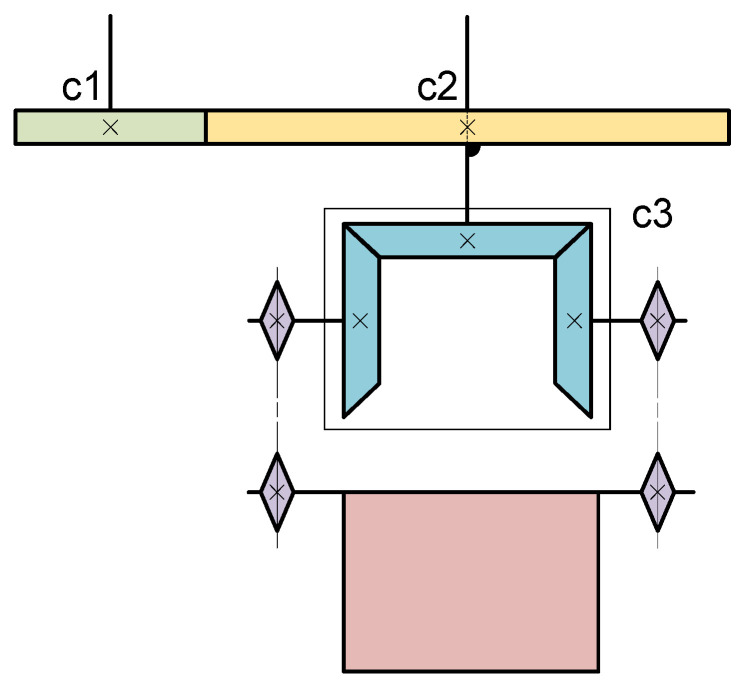
Structural principle of the discharge end.

The discharge end has two degrees of freedom: spinning and flipping. Assuming the angular velocity of the end-effector spinning is represented by $\omega_{z}$, and the angular velocity of flipping is represented by $\omega_{f}$. According to the structural schematic diagram in Figure 5, it is known that the motor driving gear c2 is a flipping drive motor, with its motor driving angular velocity denoted as follows:

(4) $$\omega_{fd}=i_{l}i_{zj}i_{fj}\omega_{f}$$

where $\omega_{fd}$ represents the driving angular velocity of c2’s motor; $i_{l}$ denotes the transmission ratio of the sprocket; $i_{zj}$ stands for the reduction ratio of the right-angle reducer c3; and $i_{fj}$ signifies the reduction ratio of the reducer connected to c2’s motor.

When the discharge end spins, it requires driving the small gear c1 to rotate the entire end-effector. Simultaneously, the differential operation of c2’s motor is necessary to ensure the relative immobility of the right-angle reducer and the connecting components with the discharge end. Therefore, the driving velocities of the two motors are represented as follows:

(5) $$\left\{\begin{array}{l} \omega_{fd}=\omega_{z}\\ \omega_{zd}=i_{xd}i_{zc}\omega_{z} \end{array}\right.$$

where $\omega_{zd}$ represents the driving angular velocity of c1’s motor; $i_{xd}$ denotes the transmission ratio between the small gear and the turntable bearing; and $i_{zc}$ signifies the reduction ratio of the reducer connected to c1’s motor.

The reduction ratios for each gear in Equations (4) and (5) are listed in Table 1.

Based on the data provided in the table, the following velocity formula can be derived:

(6) $$\left\{\begin{array}{l} \Phi_{m}=G_{m}^{H}q_{md}\\ G_{m}^{H}=\left[\begin{array}{cc} 0 & \frac{1800}{17}\\ 350 & 1 \end{array}\right] \end{array}\right.$$

where $\Phi_{m}={\left[\begin{array}{ll} \omega_{f} & \omega_{z} \end{array}\right]}^{T}$ and $q_{md}={\left[\begin{array}{ll} \omega_{zd} & \omega_{fd} \end{array}\right]}^{T}$.

#### 2.2.5. Workspace Analysis

The constraints of the parallel mechanism primarily arise from its structural design. Excluding singularities of the parallel mechanism, the main limiting factors include the range of motion of the prismatic joints, the angular limitations of the kinematic pairs, and potential interference with the moving platform.

**Figure 6:**
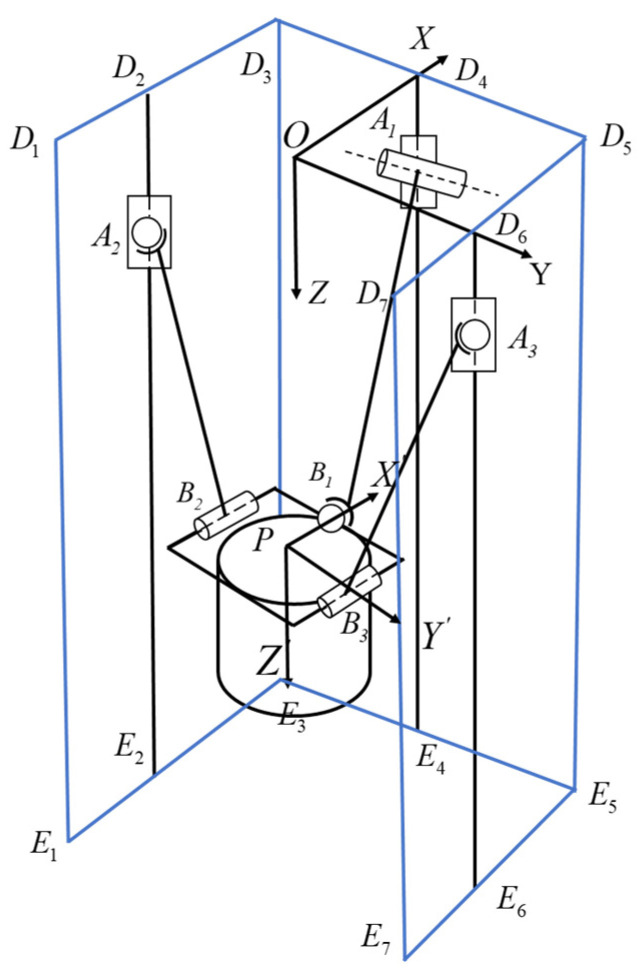
Interferometric analysis structure diagram.

As shown in Figure 6, the discharge end is modeled as a cylindrical body for analysis. This analysis reveals potential collisions between the discharge end and the supporting frame. Let the center of the lower circular surface of the cylindrical discharge end be denoted as point $P_{d}$, with the cylinder’s height as h and the radii of the top and bottom circular surfaces as $r_{P}$. Based on the end-effector’s pose, the coordinates of point $P_{d}$ can be determined as follows:

(7) $$P_{d}=(h\cos\alpha +P_{X},h\cos\beta +P_{Y},h\cos\gamma +P_{Z})$$

In three-dimensional space, a circle with center point $\left(c_{1},c_{2},c_{3}\right)$, normal vector n, and radius r can be expressed by the following parametric equation:

(8) $$\left\{\begin{array}{c} x(\theta )=c_{1}+r\cos\theta a_{1}+r\sin\theta b_{1}\\ y(\theta )=c_{2}+r\cos\theta a_{2}+r\sin\theta b_{2}\\ z(\theta )=c_{3}+r\cos\theta a_{3}+r\sin\theta b_{3} \end{array}\right.$$

where $a=(a_{1},a_{2},a_{3})$, $b=(b_{1},b_{2},b_{3})$, and a and b are two unit vectors that are orthogonal to both n and each other, while the parameter θ ranges from 0 to 2π.

Based on the parametric equation of a circle in three-dimensional space, the parametric equation of the circle on the lower plane of the discharge end is expressed as Equation (9):

(9) $$\left\{\begin{array}{c} x(\theta )=h\cos\alpha +P_{X}+r_{P}\cos\theta a_{P_{d}}+r_{P}\sin\theta b_{P_{d}}\\ y(\theta )=h\cos\beta +P_{Y}+r_{P}\cos\theta a_{P_{d}}+r_{P}\sin\theta b_{P_{d}}\\ x(\theta )=h\cos\gamma +P_{Z}+r_{P}\cos\theta a_{P_{d}}+r_{P}\sin\theta b_{P_{d}} \end{array}\right.$$

The interference conditions between the end-effector and the support frame are as follows: (1) checking the interference between the center point of the lower platform circle and the support frame; and (2) checking the interference between the lower platform circle and the support frame. These interference conditions are formulated in Equations (10) and (11). The pose of the moving platform is considered within the workspace of the parallel mechanism only if Equation (11) has no solutions and the boundary conditions of Equation (10) are satisfied.

(10) $$\left\{\begin{array}{l} h\cos\alpha +P_{X}<l_{OD_{4}}\\ l_{OD_{2}}<h\cos\beta +P_{Y}<l_{OD_{6}} \end{array}\right.$$

(11) $$\left\{\begin{array}{l} x(\theta )=c_{1}+r\cos\theta a_{1}+r\sin\theta b_{1}\\ y(\theta )=c_{2}+r\cos\theta a_{2}+r\sin\theta b_{2}\\ z(\theta )=c_{3}+r\cos\theta a_{3}+r\sin\theta b_{3}\\ x=l_{OD_{4}}\\ y=l_{OD_{2}}\\ z=l_{OD_{6}} \end{array}\right.$$

where $l_{OD_{i}}$ denotes the distance between the support frame and the coordinate system {O}.

**Figure 7:**
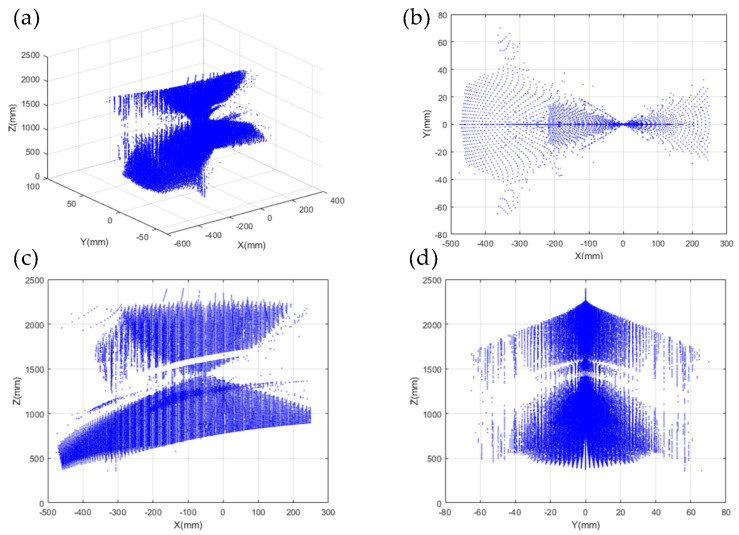
Parallel institutional workspaces: (**a**) three−dimensional workspaces; (**b**) XY orientation workspace; (**c**) XZ orientation workspace; and (**d**) YZ orientation workspace.

Based on the above analysis of factors influencing the workspace of the parallel mechanism and the parameters of the 2PSR/1PRS parallel mechanism shown in Table 2, the Monte Carlo method was applied to solve the workspace. Using MATLAB programming, the workspace of the 2PSR/1PRS parallel mechanism was obtained, as illustrated in Figure 7.

## 3. Intelligent Discharge Strategy

The requirements for the discharge robot include positioning itself above the underground tank’s opening and efficiently discharging fermented grains through the parallel mechanism. During positioning, the TOF depth camera calculates the distance between the target and the camera and generates a three-dimensional image. In the context of this discharge robot, its role is to detect the position of the tank and the surface of the fermented grains inside the tank. Once the position is detected, the XY-direction motors are driven by visual servoing to position the discharge end-effector above the tank, preparing for digging. After multiple discharge operations, the surface of the fermented grains inside the tank is detected to determine if the work is completed.

**Figure 8:**
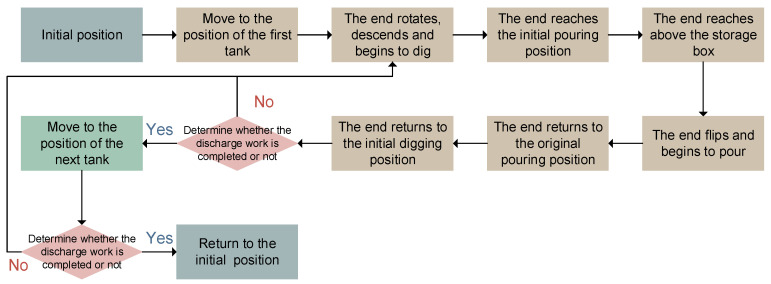
Diagram of the discharge process.

**Figure 9:**
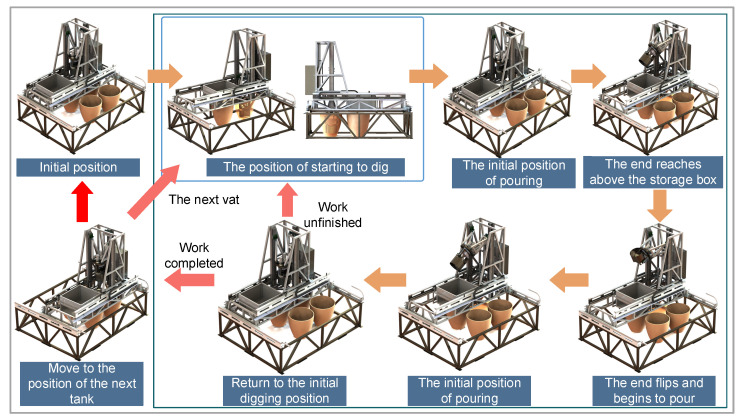
Schematic diagram of the discharge process.

The discharge process of the robot is illustrated in Figure 8 and Figure 9. After powering on, the XY-direction motors are visually located to reach the first tank position. Then, the three actuated motors of the parallel mechanism are controlled to move the parallel mechanism platform to the initial digging position. While digging, the actuated motors of the parallel mechanism are controlled to lower and rotate the discharge end to transfer fermented grains into it. Once the collection end is filled, the actuated motors position it for pouring, rotating it in the Y direction over the storage box to complete the pouring process. After pouring, the parallel mechanism returns to its initial pouring position and then back to the starting digging position. The TOF camera verifies whether the discharge for that underground tank is completed. If so, it moves to the next underground tank; if not, it repeats the process until completion. Based on this discharge process, the proposed intelligent fermented grain discharge strategy includes the perception of underground tanks’ positions, fermented grain level sensing, and motion control methods for the discharge end.

### 3.1. Improved Canny Edge Detection Algorithm

**Figure 10:**
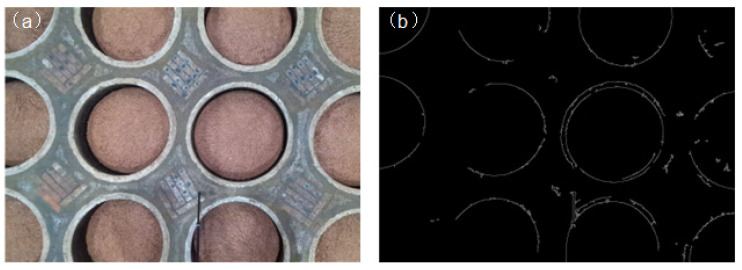
(**a**) Color images of underground tanks; and (**b**) traditional Canny test results.

The precise localization of objects in space is typically achieved through edge detection algorithms. Currently, the majority of edge detection algorithms are improvements based on the Canny operator. Traditional Canny operators utilize Gaussian filtering for noise reduction. However, Gaussian filtering lacks discriminative power. Using a larger smoothing factor achieves better noise reduction but also, to some extent, weakens edge information, leading to edge fragmentation phenomena around the tank. Conversely, using a smaller smoothing factor results in more noise in the edge image, affecting the extraction of tank edges [26]. Additionally, the dual thresholds in traditional Canny operators are manually set fixed values, which fail to meet the detection requirements of operation scenes due to variations in edge gradients caused by factors such as illumination, factory conditions, and differences of tanks. Traditional Canny operator results for underground tank detection are illustrated in Figure 10. In response to deficiencies in the Canny operator, many scholars have improved the edge detection robustness against noise in various application scenarios by employing different filtering methods [27] and thresholding techniques [28]. This paper substitutes anisotropic diffusion filtering for Gaussian filtering, thereby reducing edge blurring while maintaining a certain level of smoothness. Moreover, the automated selection of high and low thresholds based on gradient histograms is implemented to enhance edge detection effectiveness.

Anisotropic diffusion filtering, also known as PM diffusion filtering, overcomes the drawback of Gaussian filtering, which tends to blur edges, by smoothing images while preserving their edges [29]. The principle of this filtering method is to treat the image as a thermal field, with each pixel representing heat flow. Based on the relationship between the current pixel and its surrounding pixels, the decision to diffuse to the surroundings is determined. In this study, eight-directional anisotropic diffusion filtering is employed, with the formula as follows:

(12) $$\begin{array}{l} I_{x,y}^{t+1}=I_{x,y}^{t}+\lambda (c_{N_{x,y}}\nabla_{N}(I_{x,y}^{t})+c_{S_{x,y}}\nabla_{S}(I_{x,y}^{t})+c_{E_{x,y}}\nabla_{E}(I_{x,y}^{t})+c_{W_{x,y}}\nabla_{W}(I_{x,y}^{t})+\\ c_{NE_{x,y}}\nabla_{NE}(I_{x,y}^{t})+c_{NW_{x,y}}\nabla_{NW}(I_{x,y}^{t})+c_{SE_{x,y}}\nabla_{SE}(I_{x,y}^{t})+c_{SW_{x,y}}\nabla_{SW}(I_{x,y}^{t})) \end{array}$$

where $I_{x,y}$ represents the grayscale value at the pixel coordinates $\left(x,y\right)$ in the pixel coordinate system; λ is the smoothing coefficient, empirically set to 0.14 in this study; and t denotes the number of iterations, with an initial value of 0. As the number of iterations increases, the edge connections in the image improve. However, excessively high iteration numbers not only increase the computational time but also introduce noise. Considering the trade-off between detection performance and computational efficiency, this study sets t=5. The variables $\nabla_{N}(I_{x,y}^{t})$, $\nabla_{S}(I_{x,y}^{t})$, $\nabla_{E}(I_{x,y}^{t})$, $\nabla_{W}(I_{x,y}^{t})$, $\nabla_{NW}(I_{x,y}^{t})$, $\nabla_{NE}(I_{x,y}^{t})$, $\nabla_{SW}(I_{x,y}^{t})$, and $\nabla_{SE}(I_{x,y}^{t})$ represent the differential operators in the eight directions around the point.

**Figure 11:**
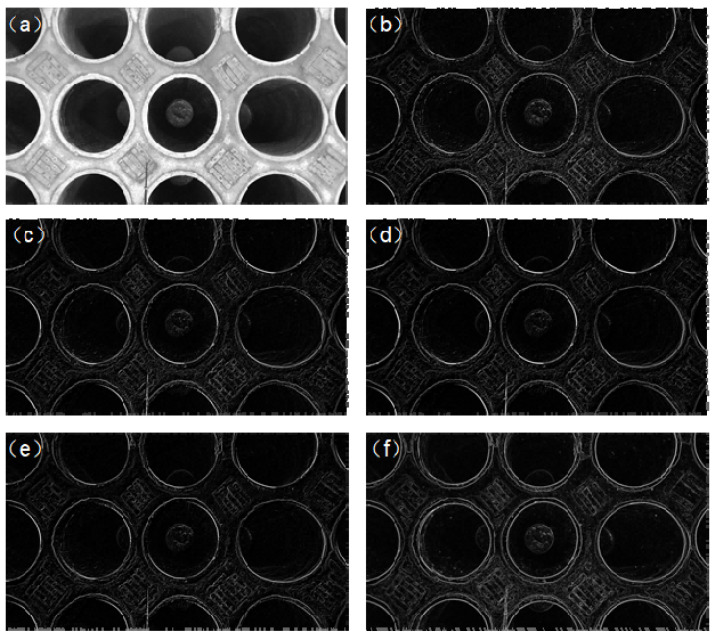
(**a**) Gray-scale map; (**b**) original image edge; (**c**) Gaussian filter edge; (**d**) mean filter edge; (**e**) median filter edge; and (**f**) anisotropic diffusion filter edge.

The filtering effects on the tank image are depicted in Figure 11. From this figure, it can be observed that, compared to Gaussian, mean, and median filtering, anisotropic diffusion filtering yields clearer edges while retaining a certain level of smoothness.

The gradient histogram of the image is used to illustrate the distribution of gradients within the image. In the gradient histogram, the majority of gradients are represented by low-frequency components, indicating minimal grayscale variations, typically attributed to object surfaces or backgrounds. Conversely, the number of pixels occupied by edges in the image is relatively small, with higher gradient values, reflected in the fewer high-frequency components in the gradient histogram [30]. Thus, based on the gradient histogram, this paper determines the magnitudes of high and low thresholds, achieving edge gradient extraction by selecting a reasonable range of gradients within the histogram. In dual-threshold detection, the low threshold is usually employed to filter out background noise, while the high threshold emphasizes edges [31]. Hence, the high threshold $TH_{1}$ and low threshold $TH_{2}$ can be set as follows:

(13) $$\left\{\begin{array}{l} TH_{1}=D_{\sigma *N}\\ TH_{2}=\alpha *TH_{1} \end{array}\right.$$

where $D_{i}$ represents the gradient magnitude of each pixel in the image, N denotes the total number of pixels in the image, $\sigma \in \left(0,1\right)$ is the proportional adjustment coefficient, $D_{\sigma *N}$ represents the gradient value at position σ∗N in the sorted gradient sequence, and $\alpha \in \left(0,1\right)$ is used to adjust the magnitude of the low threshold $TH_{2}$. The values of σ and α are determined in subsequent experiments.

**Figure 12:**
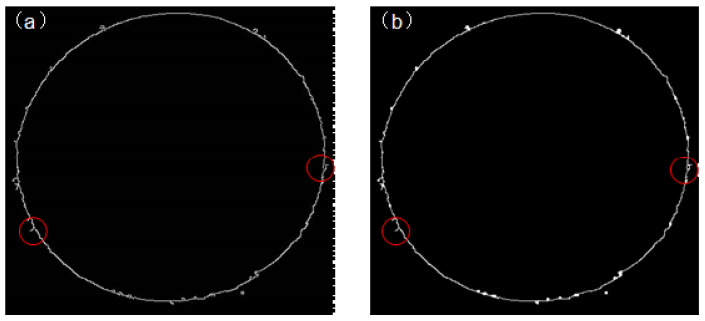
(**a**) Original underground tank edge; and (**b**) the edge of the underground tank after closing the operation (The region within the red circle highlights the changes observed before and after the closure operation.).

The edge image obtained from the improved algorithm still exhibits some flaws. Firstly, despite various measures taken to enhance the edges in areas with weak gradient intensities, small discontinuities persist, undermining the edge integrity. Secondly, the obtained edge image may retain some non-tank edge regions, affecting the determination of the tank center in subsequent processes. To address discontinuities caused by small edge breaks, a morphological closing operation is applied to optimize edge connectivity. The effect of the tank edge closing operation is illustrated in Figure 12.

**Figure 13:**
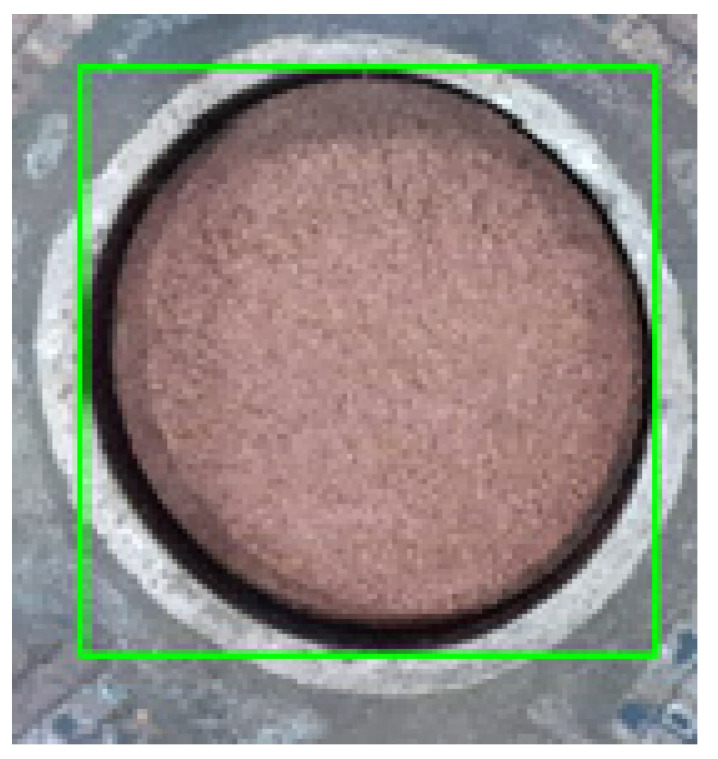
External rectangular diagram.

The acquisition of the tank center is achieved using a bounding rectangle drawn around the contour. The boundingRect function in OpenCV can draw the bounding rectangle and return the coordinates of its four points. By computation, the centroid of the bounding rectangle is obtained, which serves as the tank centroid. The bounding rectangle of the tank is depicted in Figure 13. Due to the closing operation in the image, small edges along the tank edge are connected, resulting in the phenomenon of multiple bounding rectangles for the same contour. Therefore, for bounding rectangles with centroids close to each other, the smallest rectangle in terms of size is selected as the detection result.

### 3.2. Acquisition and Processing of Fermented Grains Surface Point Cloud

During the discharging process in an underground tank, different digging motions need to be formulated based on the undulation of the fermented grains surface. Due to variations in fermentation levels, the color of the fermented grains undergoes changes, which makes it difficult to extract complete surface images using traditional image detection techniques such as color and texture. Therefore, this study utilizes the YOLO-v7 model to train a segmentation model for the fermented grains surface, enabling the acquisition of image data for the surface area. Subsequently, the corresponding color image of the surface is combined with the depth image to obtain a point cloud of the surface. Due to the large number of points in the synthesized point cloud and the computational complexity of reconstruction, downsampling algorithms are employed to streamline the point cloud. Finally, three-dimensional reconstruction is utilized to perceive the overall shape of the surface, facilitating surface perception.

#### 3.2.1. Acquisition of Fermented Grains Point Cloud

**Figure 14:**
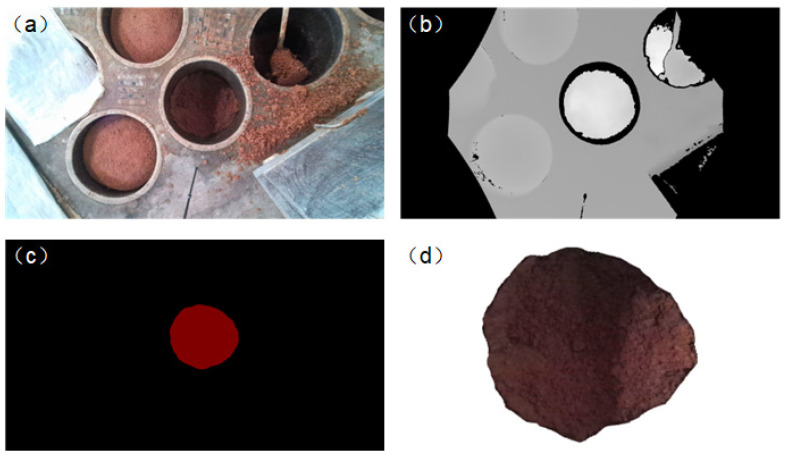
(**a**) Color image; (**b**) depth image; (**c**) mask of the fermented grains surface; and (**d**) point cloud of the fermented grains surface.

The detection results of YOLO-v7 are output in the form of monochromatic masks. These masks are then utilized to perform bitwise operations with both depth and color images, resulting in the depth and color images of the fermented grains surface. By applying coordinate transformations to the images, a point cloud of the fermented grains surface is obtained, with the color values from the corresponding color image assigned to the color channel of the point cloud. The resultant point cloud of the fermented grains surface is depicted in Figure 14d.

#### 3.2.2. Point Cloud Downsampling and Three-Dimensional Reconstruction

The synthesized point cloud data contain a large number of points that are densely distributed, resulting in high redundancy. Directly performing three-dimensional reconstruction on it would lead to excessively long reconstruction times, thereby affecting the overall operational efficiency of the equipment. Therefore, it is necessary to downsample the point cloud while retaining its shape characteristics, a process known as point cloud downsampling. The downsampled point cloud, being discrete data, cannot reflect the surface features beyond the discrete points, necessitating three-dimensional reconstruction to depict the overall shape of the surface. This study compares several common point cloud downsampling and 3D reconstruction algorithms applied to the material surface point cloud, evaluating the errors and runtime of each algorithm to select the optimal one.

The curvature-based downsampling algorithm uses the curvature values of the point cloud surface as the downsampling criterion. It increases the sampling density in high-curvature regions and decreases it in low-curvature regions, highlighting the geometric features of the point cloud while maintaining shape accuracy under the same sampling count. The voxel-based downsampling algorithm simplifies the point cloud using a voxel grid. This algorithm converts the point cloud into a voxel grid, divides it into smaller, equal-sized voxels, and calculates the centroid of the point cloud in each voxel. The centroid replaces the entire point cloud within the voxel, effectively reducing the point cloud density. The Farthest Point Sampling (FPS) algorithm aims to maximize the distance between sampling points, ensuring better coverage of the sampled points.

**Figure 15:**
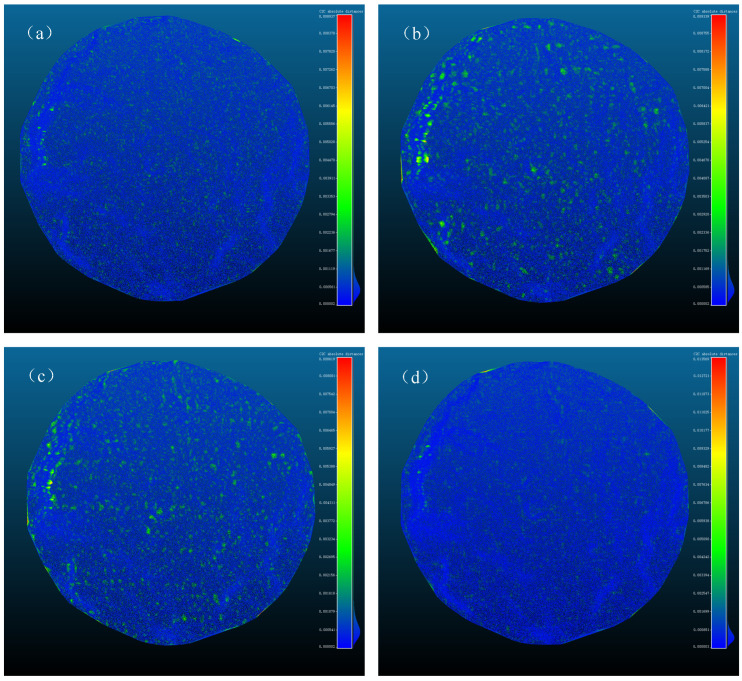
(**a**) The error graph for FPS downsampling with M = 9000; (**b**) the error graph for curvature downsampling with H = 4, L = 13; (**c**) the error graph for curvature downsampling with H = 8, L = 10; and (**d**) the error graph for voxel downsampling with W = 0.0094.

These three algorithms were applied to downsample the material surface point cloud, limiting the resulting point cloud to 8–12% of the original size. For the FPS algorithm, the sampling count was set to M = 9000. In the curvature-based downsampling, configurations H = 4, L = 13 and H = 10, L = 8 both met the requirements, where H and L represent the sampling intervals for high- and low-curvature regions, respectively. The voxel downsampling algorithm reduced the voxel size to W = 0.0094. The runtime of each algorithm under different parameters was recorded, and Delaunay triangulations of both the original and downsampled point clouds were generated using CloudCompare 2.13.alpha (7 February 2023) software to evaluate the shape preservation of each downsampling method. Since a direct comparison of the face shapes of the reconstructed models was not possible, uniform resampling was applied to the resulting models, with the number of sampling points set to ten times that of the original point cloud. The errors between the reconstructed point clouds and the original model were calculated using CloudCompare’s point cloud distance function and visualized as heat maps, as shown in Figure 15.

**Figure 16:**
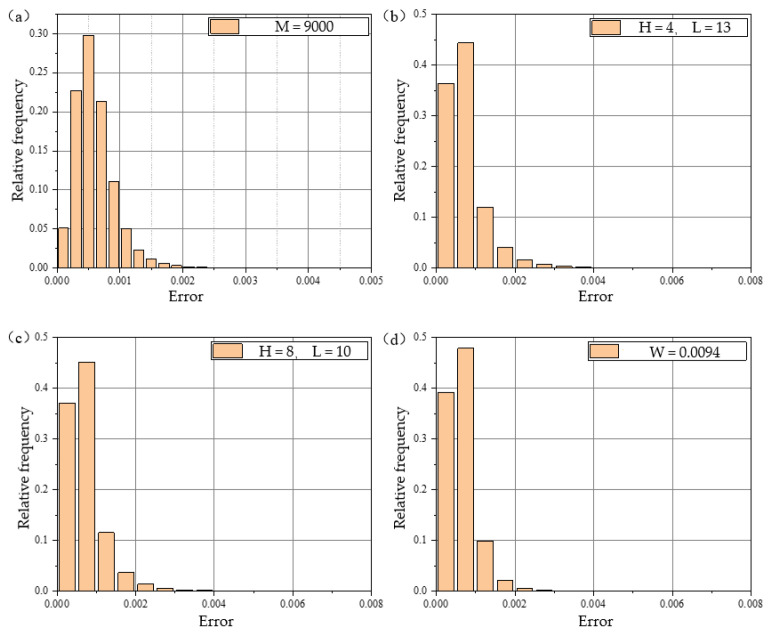
(**a**) The error histogram for FPS downsampling with M = 9000; (**b**) the error histogram for curvature downsampling with H = 4, L = 13; (**c**) the error histogram for curvature downsampling with H = 8, L = 10; and (**d**) the error histogram for voxel downsampling with W = 0.0094.

As observed from the figure, the FPS and voxel downsampling algorithms preserved the shape of the point cloud effectively. To analyze the errors visually, histograms of the errors were plotted, as shown in Figure 16, and the maximum and root mean square (RMS) errors were calculated, as detailed in Table 3. From Figure 16 and Table 3, it is evident that, when downsampling to 9000 points, the FPS algorithm produced the best sampling performance. The voxel downsampling algorithm showed slightly higher RMS errors compared to FPS, but it required significantly less time—only 0.25% of the FPS runtime. Considering both the performance and computation time, the voxel downsampling algorithm was selected as the optimal downsampling method for this study.

**Figure 17:**
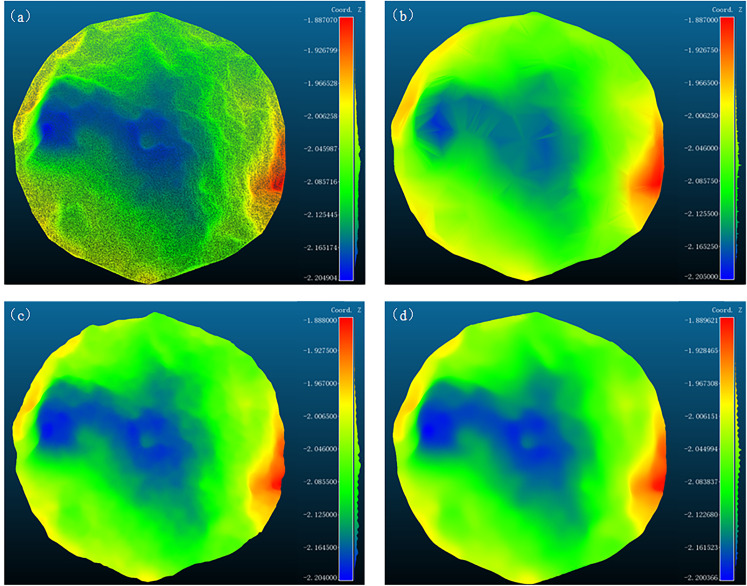
(**a**) Point cloud of material surface; (**b**) 3D reconstruction result of α-shape method; (**c**) 3D reconstruction result of BPA; and (**d**) 3D reconstruction result of Poisson algorithm.

Currently, 3D reconstruction algorithms can be categorized based on their principles into Delaunay-triangulation-based surface reconstruction, implicit surface reconstruction, and region-growing surface reconstruction methods. In this study, three typical 3D reconstruction algorithms—α-shape, Ball Pivoting Algorithm (BPA), and Poisson surface reconstruction—were selected to evaluate their performance on reconstructing the mash surface. Each algorithm was used to reconstruct the material surface, and the results were imported into CloudCompare software. Heat maps were generated based on the Z-axis values, as shown in Figure 17.

**Figure 18:**
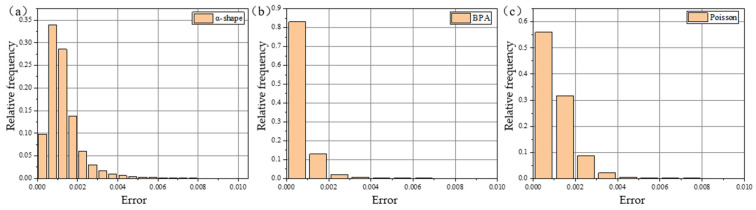
(**a**) The error histogram for α-shape method; (**b**) the error histogram for BPA; and (**c**) the error histogram for Poisson algorithm.

As shown in Figure 17, all three algorithms roughly captured the geometric features of the material surface. The ball pivoting and Poisson surface reconstruction methods provided more accurate reconstructions, with the undulations of the reconstructed models closely matching those of the point cloud. However, the α-shape method exhibited distortion at the boundaries of regions with significant undulation. To quantitatively assess the reconstruction performance, the distance errors between the reconstructed models and the initial point cloud were computed, and the error histograms are presented in Figure 18. The average errors and algorithm runtimes are summarized in Table 4. From Figure 18 and Table 4, it is clear that the ball pivoting algorithm outperforms the α-shape algorithm in both reconstruction accuracy and runtime. Compared to the Poisson surface reconstruction, the ball pivoting algorithm offers a similar reconstruction accuracy but with a significantly reduced runtime. Therefore, the ball pivoting algorithm was chosen for 3D reconstruction in this study.

### 3.3. Motion Control of Fermented Grains Based on Visual Perception

#### 3.3.1. In-Tank Space Perception

During the pouring process, collisions between the discharge end and the underground tank must be avoided, and the three-dimensional reconstruction of the surface can only reflect the distribution of the visible parts of the fermented grains. The area below the surface cannot be perceived. Therefore, it is necessary to sense the interior environment of the tank in practical discharge operations.

**Figure 19:**
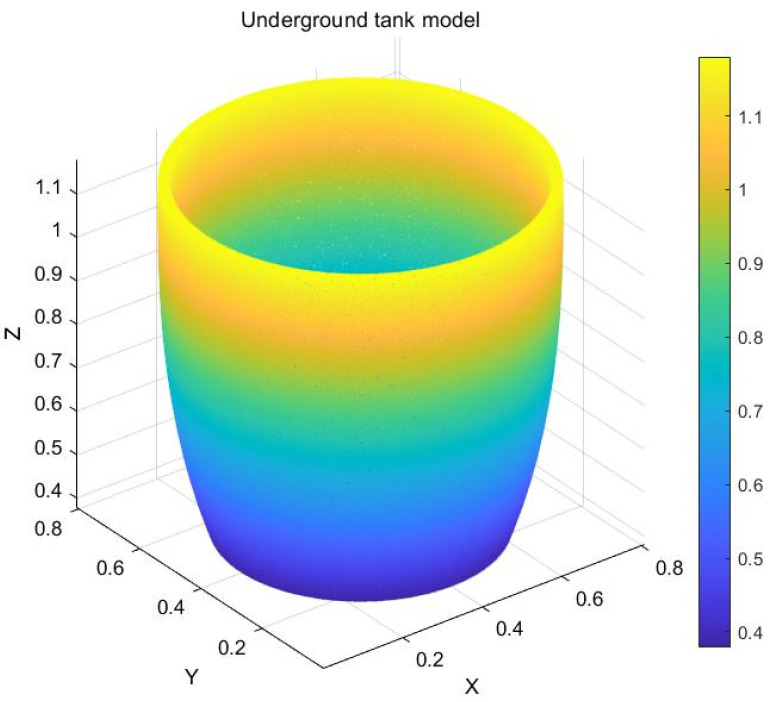
Underground tank model.

When capturing images of the underground tanks by a depth camera, the absorption characteristics of the tank walls result in severe data loss in the point cloud of the tank wall area, rendering it unsuitable for the direct perception of the interior environment. The discharge scenario addressed in this paper is based on an actual production workshop in a distillery, where the tanks used for fermentation are custom-made and manually adjusted. Since the dimensions of the tanks produced are relatively consistent, a pre-built tank model can be registered to the discharge environment, indirectly facilitating the perception of the tank. The dimensions of the tank model were obtained from the enterprise, and, combined with actual manual measurements, a 3D model of the tank was constructed using MATLAB R2020b, as depicted in Figure 19.

After obtaining the shape of the tank, it is necessary to determine the position and orientation of the tank in the detection scene. The determination of the tank’s position has been completed, and only the orientation of the tank needs to be determined. Since the tank is a rotating body, its orientation in space can be obtained from the direction of the rotation axis. The tank is vertically embedded below the ground surface in the production workshop, so the calculation of the orientation of the tank mouth can be transformed into the calculation of the normal vector of the ground plane.

This paper adopts the RANSAC method to fit the plane and obtain the normal vector of the ground plane. Random Sample Consensus (RANSAC), proposed by Fischler et al., is a method for estimating mathematical models from observed datasets using iterations. The algorithm iteratively estimates the parameters of the mathematical model from a set of observation data that may contain outliers. RANSAC is robust against noise and outliers, capable of extracting correct information from data containing noise and outliers.

**Figure 20:**
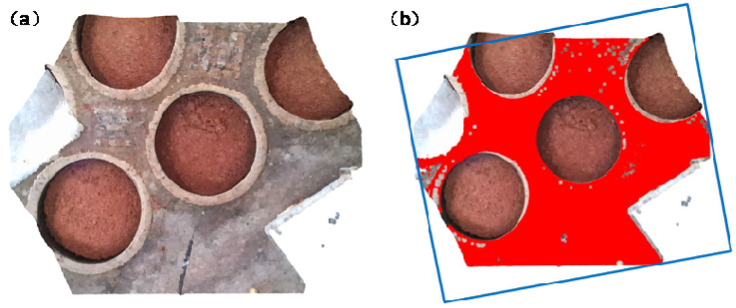
(**a**) Initial point cloud; and (**b**) plane-fitting effect.

The plane-fitting effect is shown in Figure 20, where the blue represents the fitted plane, and the red part represents the inlier set of the optimal fitted plane.

**Figure 21:**
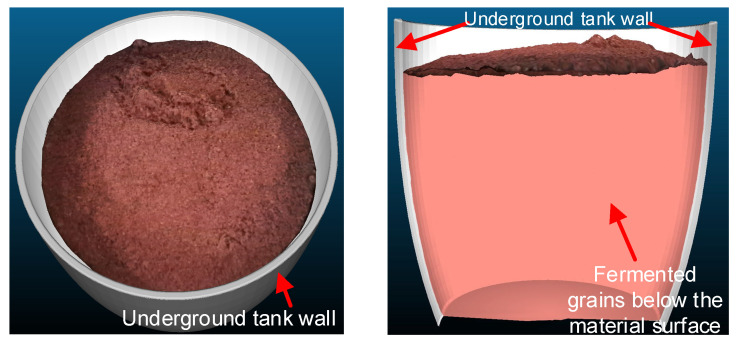
Results of the registration of the underground vat model with the point cloud of the grain surface.

For the surface shown in Figure 20, its operating range is obtained using the tank model. The registration effect of the surface with the tank model is depicted in Figure 21.

**Figure 22:**
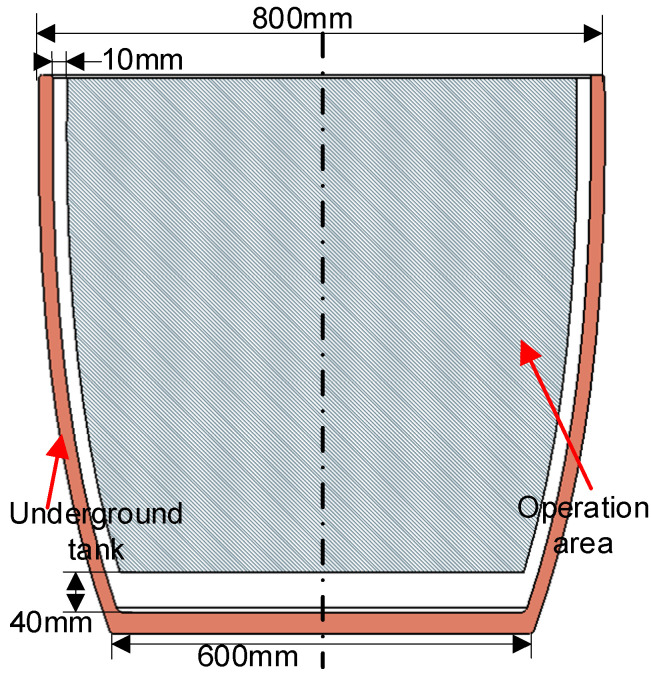
Operation area diagram.

Due to the inherent errors in tank position detection and the actual robot motion, a certain safety distance needs to be reserved at the tank wall during actual discharge operations. Additionally, there is a certain distance between the spiral blades inside the discharge end and the bottom. When the height of the fermented grains surface at the bottom of the tank is less than 30 mm, work cannot be performed. Therefore, this paper reserves a certain safety operating distance based on the tank model to construct a safe operating area, avoiding collisions between the end and the tank. The safe operating area inside the tank is illustrated in Figure 22.

#### 3.3.2. Motion Control Method of Fermented Grains

**Figure 23:**
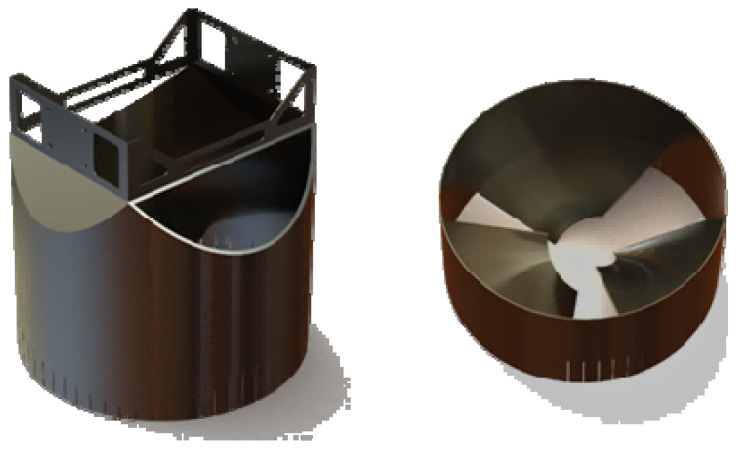
Schematic diagram of discharge end’s structure.

The structure of the discharge end addressed in this paper is depicted in Figure 23, with three sets of spiral blades at its bottom. The discharge of fermented grains is achieved through the rotational and feed movements of the end.

During the discharging process, the fermented grains exert a certain force on the discharge end. When the end is perpendicular to the fermented grains, the reactive force of the fermented grains against the end is opposite to the feeding direction, resulting in a substantial force on the parallel mechanism without causing damage to the equipment. However, when the fermented grains are inclined relative to the end, it generates a force perpendicular to the feeding direction at the bottom of the end, which can subject the end flipping device to significant impact, leading to potential damage. Therefore, the digging direction of the end should ideally be perpendicular to the fermented grains surface.

**Figure 24:**
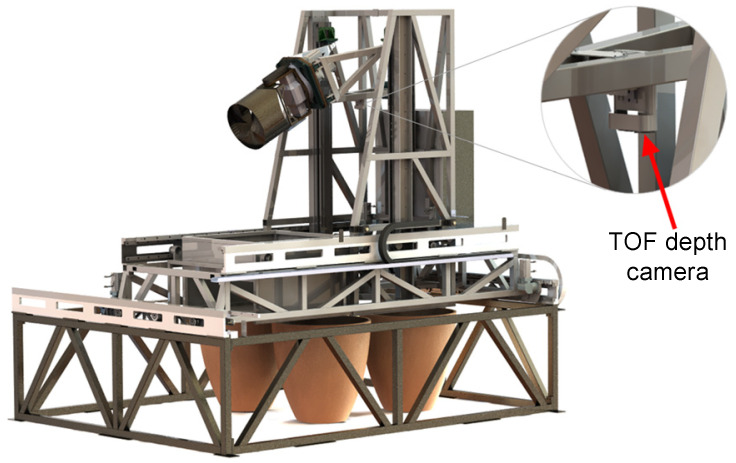
Camera-mounting position schematic.

The visual camera of the discharge robot is mounted on the parallel mechanism, with its specific installation position shown in Figure 24. From the mechanism’s position, it can be observed that the depth camera can only capture images under specific poses of the mechanism, making real-time detection during discharging unattainable. Additionally, the fermented grains tend to slide off after digging, filling the resulting void, thereby preventing the estimation of the fermented grains surface morphology post-digging. To address these issues, this study adopts a single digging approach, where image information is obtained during the pouring process after each digging cycle, informing the planning of subsequent discharge strategies.

With regard to the structural characteristics of the discharge robot and the operational characteristics of the underground tank, a visual-perception-based digging motion control method is proposed in this paper. This method dynamically adjusts the digging direction and feed distance based on the geometric shapes of the fermented grains surface, aiming to enhance the digging volume per cycle and improve the operational efficiency of the equipment. The digging motion control method primarily consists of the following components: the selection of digging positions and the acquisition of the digging direction and depth.

**Figure 25:**
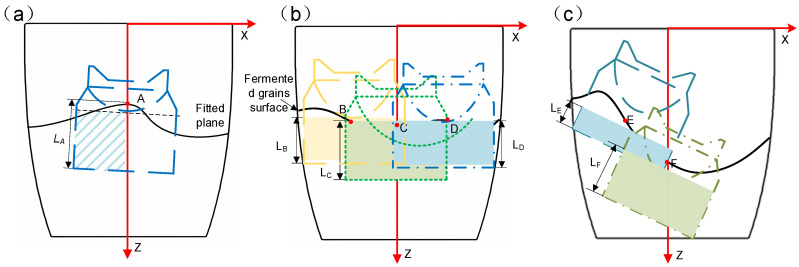
(**a**) Schematic diagram of digging with high central area; (**b**) schematic diagram of digging with gentle slope; and (**c**) schematic diagram of digging with high edge area.

The selection of digging positions: In practical operation, the fermented grains surface conditions are categorized into the following scenarios: an elevated central region, relatively flat overall surface, and raised edge region. When the central region of the fermented grains surface is elevated, as illustrated in Figure 25a, the digging work is performed at the central position A. This not only effectively reduces the height of the fermented grains surface but also enables full-load digging. After that, the fermented grains at the edge slides towards the center, minimizing significant depressions in the fermented grains surface. In the case of a relatively flat surface, as shown in Figure 25b, digging at non-central points B and D may result in the end touching the edge of the tank, preventing the digging depth L_B_ and L_D_ from reaching the maximum load-bearing depth of the end, while the central point C achieves the maximum load-bearing depth of the end, facilitating full-load digging, and, furthermore, the surrounding fermented grains fills in, resulting in a relatively flat new fermented grains surface, conducive to the continuation of digging operations. When the edge region of the surface is elevated, as depicted in Figure 25c, selecting the central point F to dig yields a greater digging volume of fermented grains compared to the edge point E, leading to a more pronounced decrease in the surface and superior efficiency.

Through the comprehensive analysis above, it can be observed that, when digging at the center of the fermented grains surface, a larger feed volume and a smaller drop in the fermented grains surface are typically achieved. Therefore, the starting point is selected as the intersection between the center of the fermented grains surface and the Z-axis of the underground tank coordinate system.

**Figure 26:**
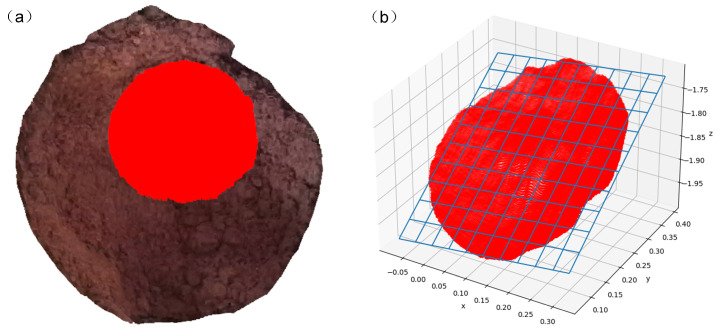
(**a**) Area to be fitted on grain surface; and (**b**) results of plane fitting.

The selection of the digging direction: To minimize the force exerted on the fermented grains surface, the digging direction should be as perpendicular to the fermented grains surface as possible. Since the actual surface is not flat, a plane-fitting method is employed to obtain the equation of the plane near the target point, with the plane normal vector serving as the digging direction. To reduce the computational complexity, a sphere is constructed at the target point with the radius of the bottom end of the end, and the nodes contained within the sphere are used for plane fitting instead of the fermented grains surface. The least squares method is employed for plane fitting, as depicted in Figure 26, where Figure 26a represents the region to be fitted, and Figure 26b shows the fitted plane result.

**Figure 27:**
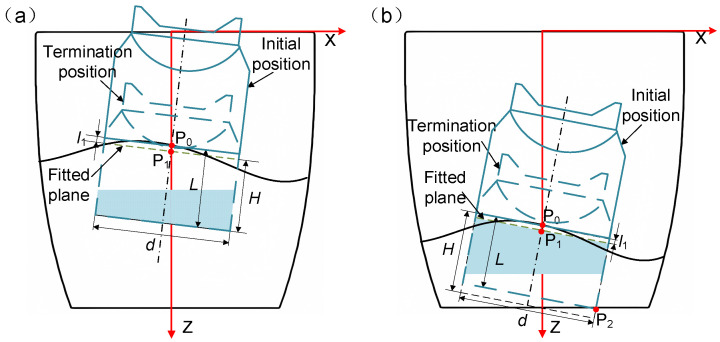
(**a**) Full digging is possible; and (**b**) full digging is not possible.

Digging depth calculation: The digging depth should be maximized for each digging direction. To achieve this, the movable distance of the discharge end along the digging direction from the starting point needs to be calculated. The specific calculation process is as follows:

Construct a cylinder with a height H using the target point $P_{0}\left(x_{0},y_{0},z_{0}\right)$ as the center of the bottom end, the diameter d of the cylinder as the bottom end diameter, and the digging direction as the axis of rotation, as shown in Figure 27.

The distance between the target point $P_{0}$ and the starting point $P_{1}$ is denoted as $l_{1}$ in Figure 27, where L represents the digging depth and $L_{d}$ denotes the actual full-load height of the discharge end. For the value of H, we assume that the end can achieve full-load digging, in which case $H=L_{d}$. If the cylinder has no intersection with the underground tank model, as shown in Figure 27a, it indicates that the end can achieve full-load digging along the current digging direction from the starting point, in which case $L=H\pm l_{1}$. If the cylinder intersects with the underground tank model, as shown in Figure 27b, it suggests that full-load digging cannot be achieved. In this scenario, the distance between each point in the intersection set and the starting point $P_{0}$ is calculated, and the point $P_{2}(x_{0}^{\prime },y_{0}^{\prime },z_{0}^{\prime })$ corresponding to the minimum distance is selected as the edge point on the bottom of the end at the farthest digging position. In this case, the formula for calculating the digging depth L is as follows:

(14) $$L=\sqrt{{\left(x_{0}^{\prime }-x_{0}\right)}^{2}+{\left(y_{0}^{\prime }-y_{0}\right)}^{2}+{\left(z_{0}^{\prime }-z_{0}\right)}^{2}-{\left(d/2\right)}^{2}}$$

## 4. Experiment

### 4.1. Improved Canny Underground Tank Edge Detection Results

**Figure 28:**
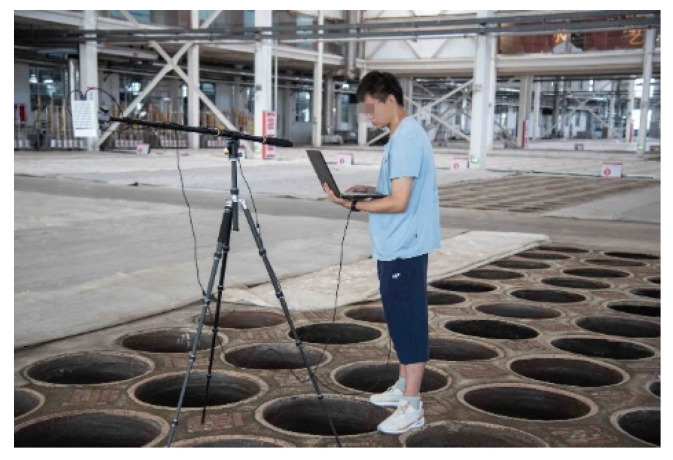
Underground tank data acquisition.

To assess the effectiveness of the improved Canny operator in detecting potholes, this study, conducted in a Windows 10 environment, employed Python programming language to implement the algorithm. Experimental images were obtained from the production workshop of a distillery in Hengshui, Hebei, China, as depicted in Figure 28. A dataset comprising 500 underground tank images from 20 different scenes was assembled for algorithm testing purposes.

**Figure 29:**
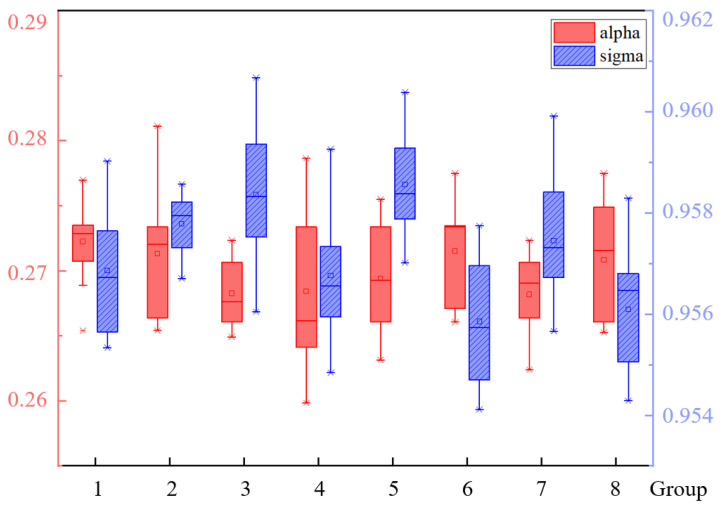
Gradient histogram experiment results.

First, coefficients for the gradient histogram method were obtained through experimental testing. Then, eight sets of data were selected, each comprising 10 images from different scenes, utilizing the improved Canny operator to extract the edges of underground tanks. High and low thresholds were manually adjusted, and corresponding values of σ and α were computed. The distribution of obtained σ and α values is illustrated in Figure 29.

From Figure 29, it can be inferred that better detection results are achieved when σ ranges between 0.954 and 0.960, and α ranges between 0.265 and 0.274. Since parameter α controls the value of the low threshold with minimal impact on overall edge detection, a fixed value of 0.27 is chosen for α to save computational time, while σ is set to 0.954.

**Figure 30:**
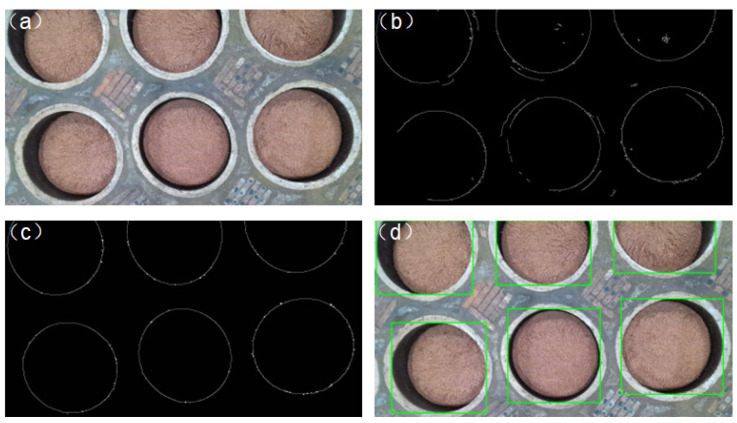
(**a**) Color image of underground tanks; (**b**) traditional Canny test results; (**c**) improved algorithm detection results in this paper; and (**d**) underground tank detection results.

To evaluate the effectiveness of the underground tank detection algorithm proposed in this paper regarding edge detection, a comparison between the traditional Canny operator and the proposed algorithm is conducted. Results are presented in Figure 30. Figure 30b depicts the edge image obtained using the traditional Canny operator, while Figure 30c illustrates the edge image acquired using the improved Canny operator proposed in this paper. Figure 30d showcases the detection results. It can be observed from Figure 30c,d that the proposed algorithm exhibits superior edge connectivity compared to the traditional Canny operator, resulting in clearer edges and fewer fragmented edges, thereby achieving more accurate pothole identification.

In order to analyze the recognition rate and accuracy of underground tank detection, 20 images were manually selected from each of eight different scenes, with the bounding box manually drawn to obtain the center coordinates. In practical operations of the discharge robot, a positioning error of less than 10 mm is required. If the pixel distance between the algorithm-obtained pothole center and the actual tank center is less than four times the pixel size (approximately 8 mm), it is considered that the algorithm-obtained pothole center coincides with the actual tank center. The recognition rates obtained from detection for each group are presented in Table 5.

**Figure 31:**
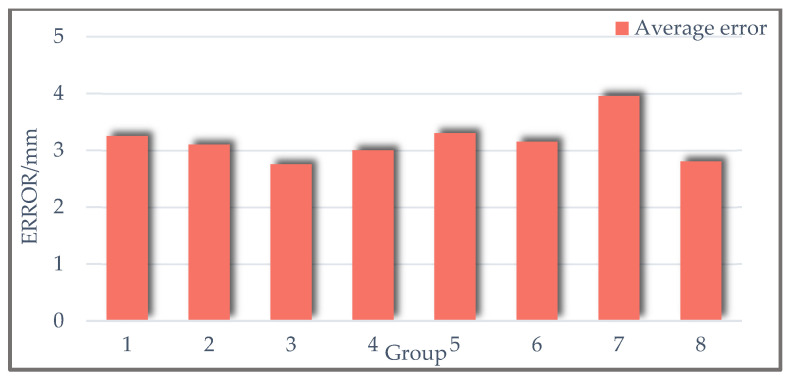
Underground tank positioning error.

To obtain the positioning error of successfully detected underground tanks in each group, the centroid coordinates of manually annotated underground tank bounding boxes were calculated using depth information. Subsequently, the error between these coordinates and those obtained by the algorithm was computed, resulting in the average error displayed in Figure 31. It can be observed from Table 5 and Figure 31 that the proposed underground tank detection algorithm based on the improved Canny operator exhibits favorable recognition rates and low positioning errors.

### 4.2. YOLO Model Training and Material Surface Segmentation

**Figure 32:**
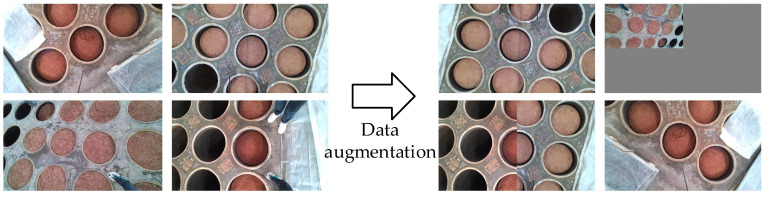
Data augmentation diagram.

The training of the YOLO-v7 model necessitates a substantial amount of image data. To acquire these data, several sets of images were collected from a distillery’s production workshop, from which 500 high-quality images were manually selected as the dataset. However, given the requirements of deep learning for a sufficient volume of image data, mere image captures were insufficient to meet the quantity demands. Therefore, image augmentation techniques such as scaling, stitching, rotating, mirroring, adjusting saturation, and adding noise were employed to expand the training data. As shown in the Figure 32, the augmented dataset comprised 1200 images, with approximately 6000 surface targets. Data augmentation enhances the diversity of samples, thereby improving the model’s generalization ability. This ensures that the augmented data maintain the same label relationships as the original data, preventing the introduction of irrelevant noise or erroneous information. Additionally, a well-designed augmentation strategy can mitigate overfitting, enhancing the model’s robustness and accuracy.

**Figure 33:**
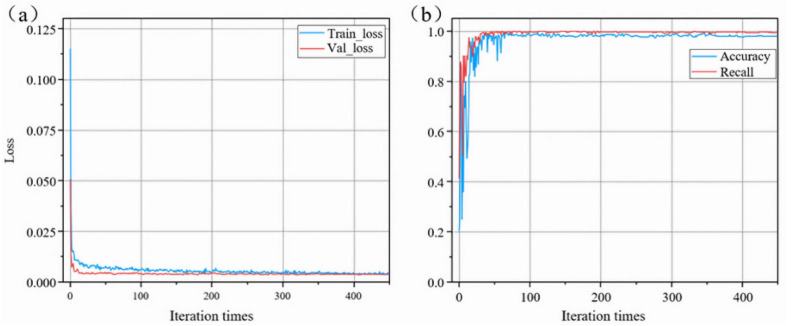
(**a**) Loss curves; and (**b**) accuracy curve and recall curve.

**Figure 34:**
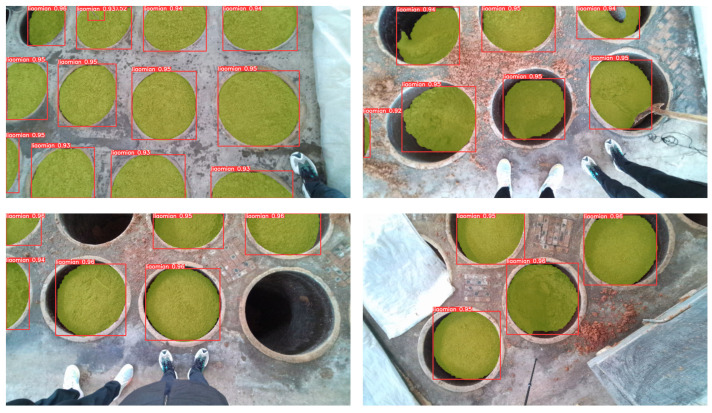
YOLO-v7 material surface test results.

Using PyTorch1.12.0 as the backend, we trained our algorithm on Windows 10. All experiments were conducted on a computer with CPU Intel Core i9-10900F@2.80GHz (Manufacturer: Intel Corporation, city: Santa Clara, CA, country: USA), GPU Nvidia GeForce RTX 4060TI (Manufacturer: Nvidia Corporation, city: Santa Clara, California, country: USA) and 128 GB memory (Manufacturer: Kingston Technology Company, city: Fountain Valley, California, country: USA). The parameters used for neural network training are presented in Table 6. The loss curves of the training and validation sets are depicted in Figure 33a. It is evident from the graph that, as the number of iterations increases, both training and validation losses steadily decrease, reaching their minimum at the 350th iteration, with training and validation losses of 0.00465 and 0.00421, respectively. Accuracy and recall curves are illustrated in Figure 33b, with the highest accuracy recorded at 0.98298 and the highest recall at 0.99415. The convergence of accuracy and recall rates in the graph is relatively rapid, possibly attributed to the utilization of pre-trained weights. The detection results of the model are illustrated in Figure 34.

### 4.3. Experimental Validation of Digging Motion Control

**Figure 35:**
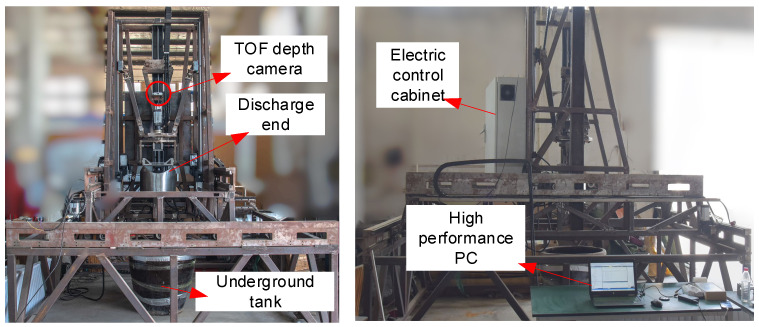
Schematic diagram of the experimental site environment.

To validate the feasibility of the proposed motion control method in this paper, an experimental platform simulating the real operational environment of the discharge robot was constructed, as depicted in Figure 35. The real-time computation of the discharge robot’s movements was implemented on a Jetson Nano. Upon receiving image data from a depth camera, the high-performance PC processed it and subsequently sent motion positions and directions to the Jetson Nano. Within the Jetson Nano, these positions and directions were converted into actual motor operating angles, which were then sequentially transmitted to the Beckhoff controller. The Beckhoff controller decoded the received communication messages, converted the motor motion parameters, and dispatched them to the nine-axis servo motors to achieve motion control of the robot.

**Figure 36:**
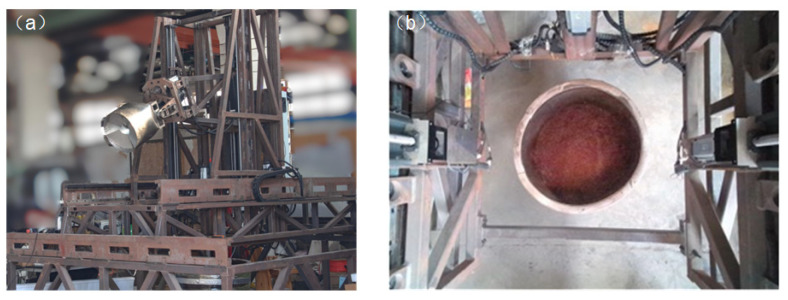
(**a**) Schematic diagram of the robot’s shooting state; and (**b**) image data capture.

Before discharging, the robot first transitions to a capturing posture to obtain image data. During this process, the intermediate guide rail of the discharge robot’s parallel mechanism descends, while the discharge end-effector extends upward. The depth camera lens faces downward, placing the robot in a photographic state. The specific posture of the robot is depicted in Figure 36a, and the captured image data are shown in Figure 36b.

**Figure 37:**
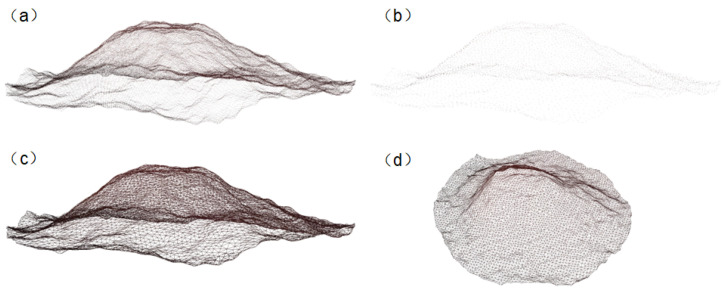
(**a**) Material surface point cloud; (**b**) voxel downsampling results; (**c**) reconstructed model by ball pivoting algorithm; and (**d**) top view of the reconstructed model.

After obtaining image data, the image mask of the fermented grains is acquired using the trained YOLO-v7 model for material surface segmentation. This mask is then combined with the color image and depth image to synthesize a point cloud of the surface. Subsequently, voxel downsampling is applied to streamline the point cloud, followed by point cloud reconstruction using ball pivoting algorithm. The results of the processing are illustrated in Figure 37.

**Figure 38:**
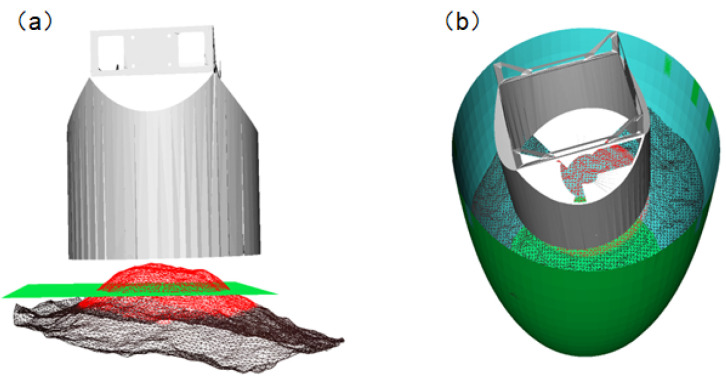
(**a**) Digging area with fitted planes; and (**b**) location of end in the operation area.

**Figure 39:**
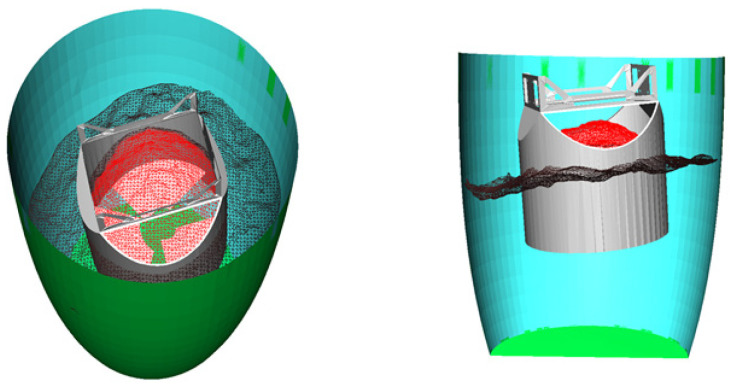
Location of end at maximum digging depth.

In the central region of the material surface, as depicted by the red area in Figure 38a, the model of the material surface to be dug is obtained. The least squares method is employed to fit this portion of the model into a plane, represented by the green section in Figure 38a. The normal vector of the plane is chosen as the digging direction. At this point, the discharge end moves above the material surface model to be dug, and the status of the material surface and the discharge end in the operational space is illustrated in Figure 38b. Subsequently, the maximum digging depth of the end is calculated assuming it can achieve full-load digging in this direction. As shown in Figure 39, the position of the end relative to the operational space confirms that, at the maximum digging depth, there is no collision with the operational area. Therefore, full-load digging can be achieved along this digging direction.

**Figure 40:**
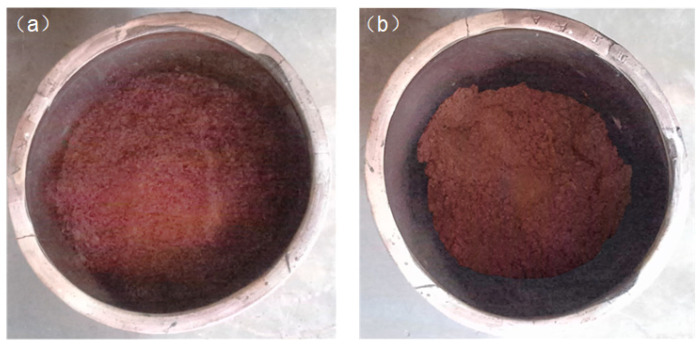
(**a**) Image before digging; and (**b**) image after digging.

**Figure 41:**
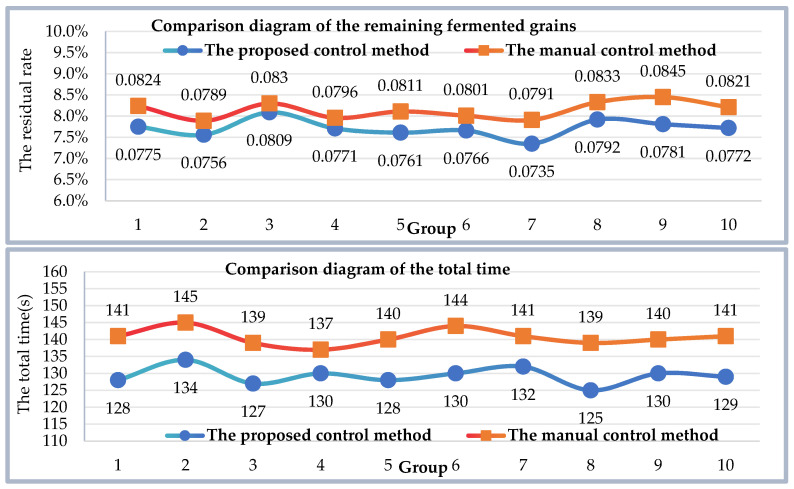
Comparison diagram of digging effect.

The single digging result of the material surface is illustrated in Figure 40. From the figure, it is evident that a significant decrease in surface height is observed after digging. To validate the efficiency of the proposed discharge control method, ten groups of equal volumes of fermented grains were set up. Each group underwent ten digs using both the proposed motion control method and manual control of the discharge robot. To address the issue of fermented grains scattering or sticking to the inner walls, each underground tank was weighed before discharging to ensure consistent fermented grains. Subsequently, the total time of dig and remaining fermented grains were recorded for each group, as depicted in Figure 41. The proposed motion control method averaged a time of 129.3 s and left approximately 7.718% fermented grains on average, whereas manual control of the discharge robot averaged 140.7 s with approximately 8.141% fermented grains remaining. Experimental results demonstrate that the proposed discharge motion control method improved the excavation speed of a single fermentation tank by 8.1% compared to manual control of the discharge robot. Taking the Hengshui distillery as an example, workers are required to complete the extraction of 56 underground tanks within 3 h. The 8.1% efficiency improvement not only reduces the likelihood of overtime work and lowers labor costs but also allows the saved time to be allocated to additional excavation tasks. This enhancement significantly increases production capacity, thereby accommodating potential future production demands and delivering notable economic and operational benefits.

## 5. Conclusions

This paper presents the overall structure and control system of a custom-developed multi-degree-of-freedom hybrid discharge robot. The parallel mechanism’s degrees of freedom and kinematics are analyzed, alongside the proposal of an intelligent discharge strategy based on visual perception. Initially, an enhanced Canny edge detection algorithm is employed to obtain the image coordinates of the underground tank opening center, which are then transformed into 3D coordinates using depth information to perceive the tank’s position. A YOLO-v7 neural network trained for fermented grains surface segmentation retrieves data images of the surface area, combining the corresponding color and depth images to generate a 3D point cloud. Voxel downsampling is applied to streamline the point cloud, followed by a three-dimensional reconstruction of the surface point cloud using the ball pivoting algorithm to obtain the overall shape of the surface, achieving surface perception. Subsequently, pre-built standard tank and surface models are matched, proposing a digging motion control method based on the models of the operating environment and fermented grains. This control method can dynamically adjust the digging direction and feed depth according to the geometric shapes of the fermented grains surface. Finally, experimental verification demonstrates that the proposed visual-perception-based digging motion control method enables the automatic operation of the discharge robot, achieving a high discharge efficiency and meeting the expected performance criteria. Currently, there are several areas where further improvements can be made in the research on perception and intelligent discharge strategies for discharge robots. First, the camera detection height in this study is relatively high. Although high-resolution images were used, the actual distance corresponding to a single pixel remains large, which limits the further enhancement of the localization accuracy. Future work could explore integrating alternative detection methods with visual sensing to improve accuracy. Second, YOLOv7 was utilized in this study to train a segmentation model for the fermentation material surface, demonstrating favorable segmentation performance. In future research, we plan to compare the performance of various neural network models and further investigate material surface segmentation based on these findings. Despite these limitations, the modular design of the robot facilitates functional expansion according to the specific requirements of different production lines. The optimization features of the intelligent system also effectively extend the equipment’s lifespan and reduce maintenance costs. In conclusion, we believe our work not only holds significant market potential but also contributes to advancing further research in this field.

## Figures and Tables

**Table 1:** Parameter table of each reduction ratio.

Name	Numerical Value
$\mathrm{Transmission}\;\mathrm{ratio}\;\mathrm{of}\;\mathrm{the}\;\mathrm{sprocket}:\;i_{l}$	1
Reduction ratio of c3 $:\;i_{zj}$	5
Reduction ratio of the gear reducer connected to c2 $’s\;\mathrm{motor}:\;i_{fj}$	70
$\mathrm{Transmission}\;\mathrm{ratio}\;\mathrm{between}\;\mathrm{the}\;\mathrm{pinion}\;\mathrm{gear}\;\mathrm{and}\;\mathrm{the}\;\mathrm{turntable}\;\mathrm{bearing}:\;i_{xd}$	120/34
Reduction ratio of the gear reducer connected to c1 $’s\;\mathrm{motor}:\;i_{zc}$	30

**Table 2:** 2PSR/1PRS parallel mechanism parameter table.

Parameters	Numerical Value
$\mathrm{The}\;\mathrm{travel}\;\mathrm{range}\;\mathrm{of}\;\mathrm{the}\;\mathrm{prismatic}\;\mathrm{joint}:\;L_{\max}$	1680 mm
The height of the cylinder: h	748 mm
$\mathrm{The}\;\mathrm{radii}\;\mathrm{of}\;\mathrm{the}\;\mathrm{top}\;\mathrm{and}\;\mathrm{bottom}\;\mathrm{circular}\;\mathrm{faces}:\;r_{P}$	400 mm
$l_{OD_{4}}$	550 mm
$l_{OD_{2}}$	534 mm
$\mathrm{The}\;\mathrm{length}\;\mathrm{of}\;\mathrm{link}\;A_{1}B_{1}$ $:\;l_{1}$	825 mm
$\mathrm{The}\;\mathrm{length}\;\mathrm{of}\;\mathrm{link}\;A_{2}B_{2}$ $:\;l_{2}$	650 mm
$\mathrm{The}\;\mathrm{length}\;\mathrm{of}\;\mathrm{link}\;A_{3}B_{3}$ $:\;l_{3}$	650 mm
The maximum rotation angle of the spherical joint	14°

**Table 3:** The result analysis of subsampling algorithms.

Algorithms	Parameters	The Number of Points	Runtime(s)	RMSE
FPS	M = 9000	9000	0.801523	0.0006908300
Curvature-based downsampling	H = 4, L = 13	9046	1.977056	0.0009401315
Curvature-based downsampling	H = 8, L = 10	9040	1.964903	0.0008853890
Voxel downsampling	W = 0.0094	9086	0.001998	0.0007709814

**Table 4:** The result analysis of reconstruction algorithm.

Algorithms	The Average Errors	SD	Runtime (s)	RMSE
BPA	0.000747	0.000733	0.088995	0.001307
α-shape	0.001306	0.000923	0.616996	0.001609
Poisson	0.000987	0.000616	0.771998	0.001162

**Table 5:** Underground tank detection recognition rate.

Group	Total Number of Underground Tanks	The Number of Underground Tanks Detected	Recognition Rate
1	83	74	0.891566
2	75	70	0.933333
3	62	54	0.870968
4	71	64	0.901408
5	57	53	0.929825
6	73	65	0.890411
7	69	61	0.884058
8	74	68	0.918919

**Table 6:** Hyperparameters setting.

Hyperparameters	Numerical Value
Batch size	10
Learning rate	0.01
Epochs	1000
Optimizer	Adam
The proportion of the training set, validation set, and test set	8:1:1

## Data Availability

The raw data supporting the conclusions of this article will be made available by the authors upon request.
